# Sarcopenia in MASLD—Eat to Beat Steatosis, Move to Prove Strength

**DOI:** 10.3390/nu17010178

**Published:** 2025-01-02

**Authors:** Dana Crişan, Lucreţia Avram, Andreea Morariu-Barb, Cristiana Grapa, Ioana Hirişcau, Rareş Crăciun, Valer Donca, Andrada Nemeş

**Affiliations:** 1 Faculty of Medicine, “Iuliu Hatieganu” University of Medicine and Pharmacy, 400012 Cluj-Napoca, Romania; crisan.dc@gmail.com (D.C.); avram.lucretia9@gmail.com (L.A.); ioanahiriscau@gmail.com (I.H.); craciun.rares.calin@elearn.umfcluj.ro (R.C.); valerdonca@gmail.com (V.D.); andrada.nemes@ymail.com (A.N.); 2 Clinical Municipal Hospital, 400139 Cluj-Napoca, Romania; 3 Regional Institute of Gastroenterology and Hepatology “Prof. Dr. Octavian Fodor”, 400162 Cluj-Napoca, Romania

**Keywords:** sarcopenic obesity, sarcopenia, MASLD, metabolic syndrome, type 2 diabetes

## Abstract

The connections between sarcopenia and various chronic conditions, including type 2 diabetes (T2DM), metabolic syndrome (MetS), and liver disease have been highlighted recently. There is also a high occurrence of sarcopenia in metabolic dysfunction-associated steatotic liver disease (MASLD) patients, who are often disregarded. Both experimental and clinical findings suggest a complex, bidirectional relationship between MASLD and sarcopenia. While vitamin D, testosterone, and specific drug therapies show promise in mitigating sarcopenia, consensus on effective treatments is lacking. Recent focus on lifestyle interventions emphasizes dietary therapy and exercise for sarcopenic obesity in MASLD. Challenges arise as weight loss, a primary MASLD treatment, may lead to muscle mass reduction. The therapeutic approach to sarcopenia in morbidly obese MASLD patients also includes bariatric surgery (BS). BS induces weight loss and stabilizes metabolic imbalances, but its impact on sarcopenia is nuanced, underscoring the need for further research. Our aim is to provide a comprehensive review of the interplay between sarcopenia and MASLD and offer insight into the most recent therapeutic challenges and discoveries, as sarcopenia is often overlooked or unrecognized and poses significant challenges for managing these patients.

## 1. Introduction

Sarcopenia encompasses both quantitative (reduction in motor unit number) and qualitative (muscle fiber atrophy) aspects of skeletal muscle loss and poses a significant burden on the healthcare system. While traditionally linked to aging, research now acknowledges sarcopenia’s potential onset at earlier stages of life. It can also be the result of secondary causes, such as malignancy, physical inactivity, organ failure, and chronic conditions. Until 2018, international guidelines have correlated the body mass index (BMI) with sarcopenia, defining it as appendicular skeletal muscle mass (ASM) divided by height (square meters), which is less than two standard deviations from the mean of a young reference group. However, this measurement underestimates sarcopenia in the obese population. Therefore, a weight-adjusted index was developed. Additionally, the Foundation for the National Institutes of Health Sarcopenia Project developed a skeletal muscle index (SMI) as an alternative to sarcopenia assessment in obese patients. Furthermore, the Sarcopenia Definition and Outcomes Consortium (SDOC) recommended incorporating weakness and slowness into the definition of sarcopenia. To this day, there is no consensus on the definition of sarcopenia due to the multiple factors that contribute to its development [1,2,3,4].

The coexistence of obesity and sarcopenia is called sarcopenic obesity (SO), and an important number of studies have tried to characterize the pathophysiological mechanisms behind this reciprocal interaction [5].

## 2. Pathophysiology of Sarcopenia in MASLD

**Figure 1:**
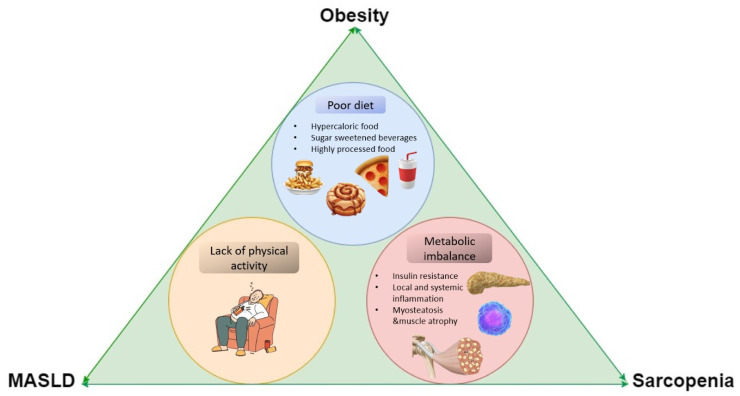
The main risk factors for obesity, MASLD, and sarcopenia.

All three elements of the obesity-MASLD-sarcopenia axis share a common pathogenetic pathway, including a lack of physical activity, poor diet, and metabolic imbalance (Figure 1).

**Figure 2:**
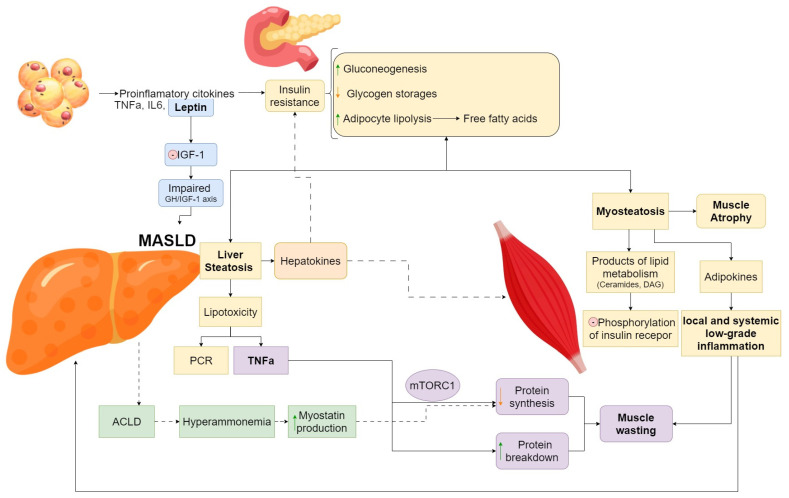
Pathophysiology of the obesity-MASLD-sarcopenia axis.

Excess adipose tissue promotes chronic inflammation by releasing pro-inflammatory cytokines, such as TNFα, interleukin-6 (IL-6), and leptin [5]. Leptin is linked to insulin resistance, hepatic inflammation, and MASLD severity. Additionally, a study correlated ASM to leptin levels, irrespective of body fat mass. Moreover, it stimulates further cytokine production and suppresses the effect of insulin-like growth factor 1 (IGF-1), disrupting the GH/IGF-1 axis, and linking sarcopenic obesity to MASLD [6,7,8] (Figure 2).

Insulin resistance leads to increased gluconeogenesis, decreased glycogen storage, and increased adipocyte lipolysis, generating free fatty acids [9]. This metabolic dysregulation promotes liver steatosis. The inability of hepatocytes to adapt to the excessive load of lipids leads to lipotoxicity, which further impairs insulin signaling and causes oxidative damage [10].

Hepatic lipotoxicity [11,12] induces the production of pro-inflammatory cytokines, such as tumor necrosis factor (TNF-α), which by altering a signaling pathway (namely, mTORC1), decreases muscle protein synthesis. It also appears to be related to the activation of other signaling cascades which promote muscle protein breakdown. These factors contribute to muscle wasting in MASLD patients [13,14].

On the other hand, an increased lipid overload in the skeletal muscle, which defines myosteatosis, is thought to impair insulin signaling through the following process: products of lipid metabolism, such as ceramides or diacylglycerols, activate intracellular kinases that inhibit the phosphorylation of the insulin receptor [15,16]. As intramuscular fat increases, there might be a false increase in muscle mass, which conceals actual myocyte atrophy and consequent loss of function. This process is not only a result of a structural change but also has a functional component, as a pro-inflammatory state is generated by adipokines and other local environmental factors, contributing to local and systemic low-grade inflammation [17]. In addition, this effect appears to be more pronounced in patients with MASH (metabolic dysfunction-associated steatohepatitis) when compared to quiescent MASLD. The effect appears even more specific, given that muscle fat content, instead of per se muscle mass, is strongly and independently associated with MASH. This indicates that myosteatosis could be either a marker for or a contributor to MASH, and MASH improvement is associated with a decrease in myosteatosis [18]. Furthermore, in fatty muscle infiltration, the local cellular homeostasis is disrupted from an energetic standpoint, in conjunction with the pro-inflammatory status. This leads to reduced protein synthesis, calcium imbalance, and subsequent decrease in the contractile function, strength, and exercise capacity [19].

Hyperammonaemia (high ammonia levels) is a common finding in advanced chronic liver disease due to hepatocellular dysfunction and porto-systemic shunting, which leads to decreased ammonia clearance. This will determine the upregulation of myostatin production, a regulator of muscle growth and size, which exerts its influence by interacting with the activin type II receptors [20]. This interaction leads to the inhibition of myoblast differentiation and the suppression of anabolic mTORC1 signaling, explaining its contribution to sarcopenia in this particular category of patients [21,22].

Some studies have demonstrated a positive correlation between a group of hepatokines (cytokines released by the liver), such as fetuin-A, hepassocin, leukocyte cell-derived chemotaxin 2 (LECT2) or follistatin, insulin resistance, and MASLD [23]. Fetuin-A seems to induce IR through various mechanisms, including the inhibition of insulin receptor tyrosine kinase in both skeletal muscle and the liver and the inhibition of insulin-sensitizing adiponectin production. Its levels were correlated with metabolic syndrome, liver fat content, and atherosclerosis [24]. Follistatin, which is broadly secreted by the liver, plays a role in the inhibition of activins or myostatins, a process that disrupts muscle growth; additionally, follistatin induces a pro-inflammatory state that promotes liver fibrosis and IR [25]. LECT2 expression in humans is linked to IR, body weight, and the inhibition of insulin signaling in myocytes [26].

Studies indicate that sarcopenic obesity is associated with increased mortality and adverse outcomes in liver cirrhosis. For instance, patients with both sarcopenia and obesity exhibit higher rates of complications and reduced survival compared to those with either condition alone [27]. However, the impact of obesity on prognosis can vary. Some research suggests that obesity alone may have a protective effect in elderly cirrhotic patients, a phenomenon known as the “obesity paradox”. Conversely, when obesity is accompanied by sarcopenia, this protective effect diminishes, leading to poorer outcomes [28]. In MASLD, the combination of increased visceral fat and muscle loss intensifies insulin resistance and systemic inflammation, accelerating liver fibrosis and elevating the risk of cirrhosis and hepatocellular carcinoma. Therefore, assessing both muscle mass and fat distribution is crucial for accurate management of patients with MASLD [29].

While the overlap in the pathophysiology of MASLD and sarcopenia is clear, the specific pathways involved in this process are yet to be elucidated. The question remains whether sarcopenia is a cause or consequence of MASLD, and additional studies are needed to accurately portray the complex interplay between these two entities.

## 3. Therapeutic Options for Sarcopenia in MASLD

### 3.1. Pharmacologic Therapies

The field of pharmacologic therapies for addressing health concerns such as sarcopenia, MASLD (Table 1), and their interrelated conditions has witnessed significant advancements and ongoing investigations. However, there are currently no approved pharmaceutical medications specifically designed for the treatment of these complex disorders.

#### 3.1.1. Vitamin D

The relationship between sarcopenia and MASLD has been established for over a decade [30]. Low levels of 25-hydroxyvitamin D (25OHD) are associated with both sarcopenia [31] and MASLD, independent of metabolic syndrome, diabetes, and insulin resistance [32]. A network meta-analysis of nine randomized controlled trials found that combining vitamin D supplementation with protein and exercise could significantly increase grip strength and muscle mass in patients with sarcopenia [33]. This suggests that vitamin D supplementation in individuals with sarcopenia might be a routine, cost-effective, and safe intervention.

#### 3.1.2. Testosterone

The reduction in testosterone and estrogen production in men and women leads to a decline in muscle mass while promoting fat production, ultimately resulting in SO. Obesity itself induces the conversion of testosterone to estradiol through the aromatization process that takes place in the adipose tissue [34].

Although testosterone supplementation presents a clear physiological rationale, the available data regarding the impact of testosterone administration on muscle function and mass remain limited and conflicting. In 2018, the task force of the International Conference on Sarcopenia and Frailty Research (ICSFR) stated that even though low testosterone levels are strongly associated with higher levels of sarcopenia [35], there was insufficient evidence to recommend anabolic hormone supplementation for older adults with sarcopenia [36].

Some studies, such as the Testosterone Trial, showed that testosterone therapy has the potential to improve metabolic factors linked to MASLD, while others report that this treatment has no direct benefit for MASLD in elderly hypogonadal men [37,38].

#### 3.1.3. Selective Androgen Receptor Modulators (SARMs)

Selective androgen receptor modulators (SARMs) selectively activate their corresponding receptors, thus decreasing the risk of adverse events [39]. SARMs were under consideration as an alternative to Testosterone Therapy for ameliorating muscle wasting associated with sarcopenia. Early preclinical models showed promise for SARMs, but recent clinical studies have raised doubts about their effectiveness. Clinical trials of Enobosarm initially showed promise for treating sarcopenia by increasing lean body mass and improving physical function. However, it ultimately failed to meet primary endpoints in phase III trials [40,41]. To this date, there are no approved medical applications for Selective Androgen Receptor Modulators (SARMs) for the treatment of MASLD and sarcopenia.

#### 3.1.4. Growth Hormones

Recent studies have emphasized the fundamental role of GH and insulin-like growth factor 1 (IGF-1) in the pathogenesis of MASH, suggesting their potential as therapeutic strategies to prevent the progression of MASLD to MASH [42,43]. In a mouse model of MASLD, Cabrera et al. revealed that IGF-1 supplementation may be a more promising therapeutic approach for MASLD compared to GH due to a safer profile and positive outcomes in reducing liver steatosis, lowering serum alanine aminotransferase (ALT) levels, and improving muscle health [44].

A study by Brioche et al. [45] demonstrated that GH replacement therapy in aged rats prevented sarcopenia by improving protein balance and enhancing antioxidant defenses. This suggests that GH therapy could mitigate muscle wasting associated with sarcopenia. Additional research indicates that lower levels of GH and insulin-like growth factor 1 (IGF-1) are associated with reduced skeletal muscle mass in the elderly. Interventions aimed at increasing GH/IGF-1 levels may help preserve muscle mass, potentially benefiting sarcopenic patients [46]. Further information regarding the impact of GH therapy in sarcopenic patients with MASLD is required.

#### 3.1.5. Ghrelin Agonists

Anamorelin, a ghrelin receptor agonist, is known for improving appetite and increasing serum insulin-like growth factor-1. It has gained approval in Japan for addressing cancer cachexia in specific cancer types [47]. A phase III trial showed that anamorelin significantly improves lean body mass and appetite in patients with cancer anorexia-cachexia syndrome [48]. However, its potential therapeutic role in NAFLD-associated sarcopenia remains unexplored.

#### 3.1.6. Drugs Targeting Myostatin and Activin Receptor Pathway

In advanced chronic liver disease patients, sarcopenia is primarily exacerbated by the upregulation of myostatin due to hyperammonaemia [49]. Consequently, targeting myostatin inhibition holds promise as a potential treatment for sarcopenia in CLD patients [50].

A single dose of Bimagrumab, an agent that interferes with the activity of myostatin, rapidly increases thigh muscle volume and total lean body mass, with no change in muscle strength, and also decreases body adiposity in older adults [51]. Another study in adults with type 2 diabetes and obesity revealed that bimagrumab enhances muscle mass and insulin sensitivity [52]. Other studies found no significant difference between participants treated with Bimagrumab and those receiving a placebo among older adults with sarcopenia who had six months of adequate nutrition and light exercise.

#### 3.1.7. Ammonia-Lowering Treatment

A preclinical study showed that ammonia-lowering treatment significantly increased lean body mass and improved grip strength and skeletal muscle growth [53]. L-ornithine L-aspartate can be an adequate option for patients with cirrhosis suffering from hepatic encephalopathy through the improvement of skeletal muscle growth and function [54]. Other recent studies have revealed that L-carnitine supplementation is linked to the mitigation of muscle loss in cirrhotic patients and with the improvement and delay of progression of MASLD patients [55,56,57]. We need further research regarding the efficacy of this supplementation in sarcopenia associated with MASLD.

#### 3.1.8. Metformin

A study by Tezze et al. demonstrated that a combination drug containing metformin and galantamine improved muscle quality in mice, implying benefits for treating sarcopenia. There is emerging evidence suggesting a potential protective role of metformin against both frailty and sarcopenia, although results vary across studies [58]; targeted studies on MASLD sarcopenic patients are required.

#### 3.1.9. Weight Loss Medication

None of the six FDA-approved medications for weight loss, including liraglutide, lorcaserin, topiramate, phentermine, bupropion, and orlistat, are approved for use in adults over 65 years, because of their mixed impacts on lean mass and bone density [59]. A study from 2018 reports that liraglutide promotes weight loss and metabolic improvements, while helping preserve lean mass in non-diabetic individuals [60]; however, the impact of weight loss medication in MASLD sarcopenic patients is not yet known.

**Table 1:** Pharmacological therapy in sarcopenic patients.

Medication	Mechanism of Action	Evidence	Sources
**Vitamin D**	Enhances calcium and phosphate metabolism, improving muscle function.	Improves grip strength and muscle mass when combined with protein and exercise.	Hong et al. (2014) [30], Scott et al. (2010) [31], Barchetta et al. (2011) [32], Badarin et al. (2021) [33]
**Testosterone**	Replenishes testosterone levels to promote muscle growth and reduce fat accumulation.	Limited and conflicting data; ICSFR does not recommend its use due to insufficient evidence.	McKee et al. (2017) [34], Dent et al. (2018) [35], Jaruvongvanich et al. (2017) [36], Lee et al. (2023) [37]
**SARMs**	Selectively activates androgen receptors, reducing adverse effects compared to testosterone.	Enobosarm showed initial promise but failed phase III trials; no approved applications.	Narayanan et al. (2018) [39], Dobs et al. (2013) [40], Crawford et al. (2016) [41]
**Growth Hormones (GH)**	Modulates GH/IGF-1 axis for muscle growth and reduced liver steatosis.	Promising preclinical and clinical studies; improved protein balance and reduced liver damage in mice; human evidence limited.	Koehler et al. (2011) [42], Cristin et al. (2023) [43], Cabrera et al. (2018) [44]
**Ghrelin Agonists**	Increases appetite and serum IGF-1 levels.	Anamorelin approved for cancer cachexia in Japan; potential in sarcopenia associated with NAFLD not yet explored.	Ebner et al. (2020) [47]
**Myostatin Inhibitors**	Blocks myostatin and activin receptor pathway to enhance muscle growth.	Bimagrumab improved lean body mass but not strength in trials; mixed results in older adults.	Kim et al. (2021) [49], Trendelenburg et al. (2009) [50], Rooks et al. (2020) [51]
**Ammonia-Lowering**	Reduces hyperammonemia and myostatin levels, improving muscle protein synthesis.	L-ornithine L-aspartate and L-carnitine linked to improved muscle growth and function in cirrhotic patients; more research needed for MASLD-related sarcopenia.	Kumar et al. (2017) [53], Butterworth (2019) [54], Allen et al. (2021) [55], Zakharova et al. (2023) [56], Savic et al. (2020) [57]
**Metformin**	Activates AMP-activated protein kinase (AMPK), improving mitochondrial function and reducing inflammation.	Shown to enhance muscle quality in preclinical studies; potential protective role in sarcopenia remains under investigation.	Pyrgioti et al. (2024) [58]
**Weight Loss Drugs**	Reduces weight by targeting appetite and metabolism; mechanisms vary among drugs.	Liraglutide preserves lean mass during weight loss in non-diabetics; effects in MASLD-related sarcopenia unknown.	Batsis et al. (2018) [59]

#### 3.1.10. Other Emerging Mechanisms and Therapeutic Potential for Sarcopenic Obesity Are Shown in the Table Below [61] (Table 2)

### 3.2. Lifestyle Intervention—Dietary Therapy

If sarcopenic obesity means a decrease in LBM and an increase in FM, as the first definition of Heber states, therapy would have to target these pathogenetic features. Treatment in sarcopenic obesity related to MASLD should target the organs involved in this complex cross-talk: adipose tissue, liver, and muscle. The aim would be to re-establish the balance among the quantity of fat tissue, liver steatosis (but also inflammation and maybe fibrosis), as well as skeletal muscle function and mass.

Approaching each of them in a thoroughly individual manner is impossible, but for theoretical and teaching objectives, we tried to separate them pathogenetically. However, decreasing adipose tissue goes hand in hand with liver steatosis treatment; for the time being, the most important recommendation for MASLD treatment is weight loss, which can be achieved the easiest way, through diet accompanied by physical activity. On the other hand, when losing weight abruptly, there is a high chance of also losing muscle mass, which is not a desirable result in our patients with severe obesity and MASLD who are already sarcopenic. That is why adopting a standard diet in these patients is very difficult.

Most of the researchers in this field are keeping the recommendations from MASLD treatment of losing 7–10% of weight to improve liver steatosis, as a weight loss of more than 7% improves the histological features of MASH [68]. Still, in patients who are probably having sarcopenic obesity, the diet should be mild and adopted in a tapered manner [69], even if the effect of diet in liver steatosis is “dose-dependent”, meaning that the more you lose weight, the more you improve your fatty liver.

Diet can assume the decrease in calories taken together regardless of macronutrients, or it can address the low intake of some of the macronutrients, especially carbohydrates or fats. The study by Kim et al. showed that a high intake of wheat gave a two-fold increase in the prevalence of sarcopenic obesity in a group of patients with cardiometabolic diseases. In an interesting manner, they obtained the same results for the meat intake, suggesting that animal proteins are not the best choice when speaking about sarcopenic obesity [70]. In the same line, it has been reported that the lacto-ovo-vegetarian dietary pattern was associated with a lower risk of SO [OR 0.79, 95% CI (0.65, 0.97); *p* = 0.027], while the meat-fish and junk food dietary patterns were not significantly associated with the risk of SO [71].

Fat intake limitation as being important in patients with SO was confirmed by a study that evaluated the role of the DASH diet, a low-fat diet, rich in fruits and vegetables and limitation in sweetened beverages and sugars. Even after controlling for total energy intake and physical activity, the negative association between DASH diet SO remained significant (OR = 0.20, 95% CI = 0.05 to 0.77, *p* = 0.01) [72].

Generally speaking, a hypoenergetic diet (>500 kcal deficit per day) results in a decrease in hepatic fat load [73].

The first observation in guidelines (EASL, Japanese, AGA [74,75]) regarding lifestyle and diet when treating MASLD was the restriction of alcohol consumption to zero as well as avoiding fructose and sucrose, as these were related to MASLD development and progression. A 50% decrease in fructose intake will improve liver steatosis, the level of aminotransferases, BMI, and glucose metabolism [76].

All the international societies for managing liver diseases recommend weight loss as the cornerstone of treating MASLD. Still, they admit that sarcopenic obesity and alcohol consumption are the most difficult hidden aspects of this problem.

Mediterranean diet seems ideal for weight loss in MASLD patients, through its balanced composition of quality proteins and fibers. The positive effect of the Mediterranean diet on MASLD was confirmed in a meta-analysis that showed a decrease in fatty liver index and also in insulin resistance [77].

As the Mediterranean diet proved to have many benefits on liver steatosis and metabolic syndrome, researchers tried a better version of the so-called ”green-rich” Mediterranean diet, involving green tea and a green shake added to the classical Mediterranean diet. The comparison between the new green version and the old one showed even better results for adding greens when searching for intrahepatic fat decrease (−38.9% vs. −19.6%; *p* = 0.035), even if both study groups lost significant weight after dieting [78].

Related to the type and amount of proteins recommended for patients with MASLD and especially those who are also present with SO, the ketogenic diet appears to provide good results for weight loss and a high amount of proteins that would promote muscle gain and function. A ketogenic diet assumes a low intake of carbohydrates (<130 g/day), but it can meet variate forms like a very low caloric ketogenic diet (<800 kcal/day, <50 g carbohydrates/day) or high-fat ketogenic diet—the classical one (unrestricted fat intake, but less than 20–50 g carbohydrates/day). The ketosis effect of these diets seemed to be beneficial for weight loss and also for MASLD improvement, irrespective of the type of ketogenic diet, but the study groups were small, and the follow-up was short, so data have to be further analyzed on larger populations [79,80].

The importance of proteins has to be highlighted when discussing sarcopenia, as a meta-analysis showed that increasing protein intake in patients depending on their age (≥65 years: 1.2–1.6 g of protein/kg/day and <65 years: ≥1.6 g of protein/kg/day) added to training with resistance exercise, increased lean body mass and muscle strength of the lower body [81]. However, another meta-analysis showed that the best strategy to promote weight loss and to gain muscle mass and strength is to combine energy restriction of 500–1000 kcal/d) with high protein intake (1.1–1.7 g of protein/kg/d) and adding to diet physical activity (combined training). Data from interventional studies are scarce regarding targeting the most helpful diet and the amount of nutrients recommended in sarcopenic obesity associated with MASLD. Until new data, the experts in this field promote protein consumption at the limit of 1.1–1.7 g/kg/day) [82]. Regarding the quality of proteins, considering that liver disease is not less important than obesity, the experts promote plant and dairy proteins over red meat [83,84,85].

It is important to underline that increased protein intake without sport will not favor the development of muscle mass. Additionally, a high amount of protein can result in an increased risk for IR and T2DM development [86].

Limited data on the subject thus suggest that weight loss in a mild and balanced manner and increased adherence to the Mediterranean diet seem to be the most beneficial in managing patients with MASLD and SO. An unbalanced diet, rich in saturated fats but also in fructose and sucrose, will promote MASLD and obesity (both subcutaneous and visceral). Adding proteins to prevent muscle mass and catabolism is also essential.

### 3.3. Lifestyle Intervention—Physical Therapy

The primary objective in treating MASLD is achieving weight loss through a combination of diet and exercise [87,88].

Physical activity, specifically structured exercises, provides benefits independent of weight loss and is a fundamental treatment for patients with MASLD. Aerobic and resistance training effectively reduces hepatic steatosis and decreases the cardiovascular risk associated with MASLD [89].

Research has shown that regular exercise triggers the secretion of insulin-like growth factor 1 (IGF-1) and decreases inflammatory cytokines such as interleukin-6 (IL-6), reactive oxygen species (ROS), and myostatin. This dual action helps prevent the progression of sarcopenia [90,91]. Consistent physical activity is not always possible for patients with physical mobility difficulties. In such cases, blood flow restriction (BFR) exercises are recommended as a training method that can achieve comparable or superior exercise outcomes using very light weights compared to traditional resistance exercises. Blood flow restriction exercises involve using a pneumatic cuff to restrict blood flow proximally to the muscle being trained, allowing individuals with limited physical mobility to perform resistance exercises at low intensity, high repetitions, and short rest intervals [92].

An important link between MASLD and sarcopenia is related to the level of physical activity. In a study of 4611 participants regarding the contribution of sarcopenia and physical inactivity to mortality in MASLD, sarcopenia was inversely related to increased physical activity level [93]. Although sarcopenia and physical deconditioning have been reported in patients with end-stage liver disease, the association with MASLD with early liver disease has also been reported [94,95].

A systematic review and meta-analysis encompassing seven randomized controlled trials (RCTs) on MASLD patients with sarcopenia revealed improved physical function with endurance or combined training. However, there was no evidence of improvement in muscle mass, and none of these studies evaluated muscle strength [96].

Yang et al. [97] provided the first investigation to suggest both resistance exercise and aerobic physical activity could exert a synergistic preventive association for MASLD in much of the general Korean population. The study utilized a nationwide representative database in South Korea to explore the independent and combined impact of aerobic physical activity and resistance exercise on MASLD. The survey encompassed 14,977 participants, revealing that highly active aerobic physical activity (at least 150 min/week) significantly reduced the risk of MASLD in both men and women [98]. Additionally, engaging in resistance exercise for ≥5 days per week was associated with a decreased risk of MASLD in men, although such a decrease was not observed in women. However, when investigating the interaction between the level of aerobic physical activity and the frequency of resistance exercise, the risks for MASLD showed a significant decrease when both types of exercise were performed more frequently in both sexes. These findings suggest that the preventive association with MASLD is notably stronger when aerobic and resistance exercises are performed together.

Lifestyle interventions prove highly effective in addressing MASLD across its spectrum, providing a comprehensive approach to managing both liver health and cardiovascular and metabolic well-being. While altering lifestyle behaviors can pose challenges for patients, personalized support facilitates significant and enduring changes. Further exploration is necessary to identify the optimal nutritional therapy combined with physical activity/exercise that enhances liver health in patients with MASLD and sarcopenia.

### 3.4. Additional Treatments—Bariatric Surgery

Bariatric surgery (BS) has become a staple in treating severely obese patients. Solid evidence shows that BS expands its benefit beyond inducing long-term weight loss, as it leads to better control of insulin resistance and diabetes remission and reduces cardiovascular events, cancer risk, and even all-cause mortality [99,100,101,102,103]. The impact of BS on sarcopenia, however, whether positive, neutral, or detrimental, is frequently overshadowed by the primary goals mentioned above. Moreover, the extent of the benefits compared to alternative therapies might be slightly overestimated, given that this method is mainly used on a population of greater risk and comorbidity burden, and thus, with the most significant potential for improvement. In MASLD patients, studies have shown that BS leads to an important reduction in major adverse liver-related outcomes [104], long-term resolution of MASH, and fibrosis regression [105,106], regardless of the surgical technique [107], being significantly more effective compared to other therapeutic approaches [108]. Despite its proven benefits, there are concerns about whether muscle mass is also affected in the weight loss process induced by BS.

The direct impact of BS on the incidence of sarcopenia is challenging to assess due to the lack of a control group. To this point, only one study has compared women at two years post-BS, at a stable weight state, to a matched, non-operated cohort. The prevalence of sarcopenia was higher in the BS group (28.3% vs. 16.6%), although not statistically significant (*p* = 0.12). However, the functional capability was apparently unaffected, as there were no differences regarding physical strength and performance [109]. These data suggest that while there might be a higher risk of post-operative sarcopenia, its functional relevance is insignificant, and the potential benefits of BS far outweigh its impact.

Data derived from a large-scale retrospective study, which collected preoperative, 1-year, and 5-year follow-up measurements of sarcopenia and frailty, identified a prevalence of 15.6% for class 1 low skeletal muscle mass (LSMM) and 4.6% for class 2 LSMM, with a slight increase in the prevalence at 5 years (16.6% for class 1 and 6.3% for class 2). The independent factors predicting LSMM were preoperative sarcopenia and age [110]. These figures are consistent with another report, which indicates that preoperative sarcopenia is a crucial determinant of postoperative nutritional status [111]. However, these studies did not consider preventive interventions and rehabilitation strategies, which might improve long-term nutritional status.

Moreover, preoperative sarcopenia seems to be a predictor for immediate therapeutic success and procedural complications, as patients with computed tomography-documented sarcopenia had a substantially higher risk of postsurgical gastric leaks (9% vs. 2%, *p* = 0.026, on a series of 205 patients) [112]. These findings suggest that a systematic assessment of sarcopenia before surgery is beneficial, as it improves patient selection, preconditioning, and short-term follow-up strategies.

Physical inactivity and a sedentary lifestyle are both a cause and a consequence of obesity, as part of a key vicious cycle for this population. Therefore, a lack of muscle conditioning through physical exercise contributes to poor muscular function. On the other hand, adequate physical exercise regimens cannot be implemented due to the other consequences of morbid obesity [113]. This epistemological rationale suggests that BS can create the premises of muscular reconditioning and regain of function through various indirect effects, as summarized in Table 3. However, these benefits might have little consequence on muscle function without a multimodal supportive approach comprising physical activity and adequate nutritional intake, as is the case for all the therapeutic methods previously discussed in the article.

In conclusion, BS is an effective therapeutic solution for patients with obesity and MASLD and significantly impacts muscle mass and function. While BS does not provide a substantial improvement in sarcopenia as a standalone intervention, it generates the premises for muscular rehabilitation through its indirect effects on weight, metabolic imbalance, and pro-inflammatory status. Used in conjunction with other lifestyle interventions as a jumpstart to therapy rather than as a quick fix, BS represents a key component in managing morbidly obese patients with sarcopenia and MASLD.

**Figure 3:**
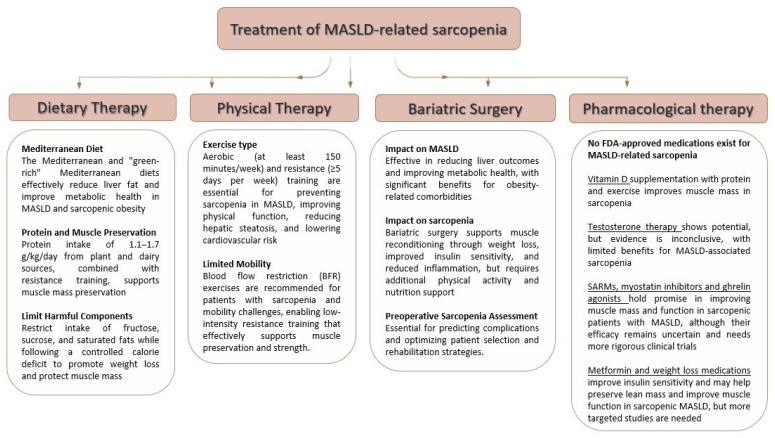
Summary of MASLD-related sarcopenia treatment.

A summary of MASLD-related sarcopenia treatment is represented in Figure 3.

## 4. Adherence to Treatment—The Gaps in Therapeutic Interventions

The cornerstone of MASLD and sarcopenia treatment is represented by lifestyle modifications, as stated. However, in the real world, adherence to lifestyle changes as imposed by healthcare providers, is far from ideal, leading to a factual ineffective treatment strategy for this particular category of patients. Loss of contact and follow-up with these patients after diagnosis decreases their chances of achieving their proposed goals, thus rendering all research on this subject obsolete if patients are non-compliant and do not benefit from proper treatment.

A prospective study on 293 histologically proven MASH patients revealed that after 52 weeks following a weight loss program based on lifestyle modifications, only 30% (88) of patients lost >5% of their weight [114]. There is limited research on factors that could potentially influence patients’ adherence to treatment, such as their perspectives, needs, and preferences; understanding these issues could improve their quality of life and, subsequently, their treatment adherence.

A qualitative bulletin board study on MASH patients, focusing on their experience and unmet needs, provided key insights into this particular category: patients generally have a minimal understanding of their disease, which mainly was found incidentally, due to its lack of symptomatology; 50% of patients do not perceive this disease has an impact on their social life or work performance in the earlier stages and they consider that their comorbidities such as diabetes or obesity are more disquieting that NASH. Patients also highlighted their need for continuous support from their healthcare providers regarding their diagnosis and treatment course and a continual support system concerning their journey through lifestyle modifications. This study emphasized the need for education as a critical aspect of increasing treatment efficiency [115]. Another important topic focused on the lack of support from healthcare providers regarding medical information as well as emotional support, emphasizing the importance of physicians providing adequate regular check-ups and motivational care.

Another qualitative study that used the Health Action Process Approach (HAPA) model revealed key barriers to adherence to lifestyle modifications prescribed to patients with MASLD. The primary discovery of this study was that most patients were aware that their main focus should be on lifestyle changes but were unsure of the specific actions needed to implement these changes. Patients need realistic action plans and coping strategies as well as support systems and action control (e.g., monitoring diet and physical activity progress by a healthcare provider) to achieve their goals [116].

A review focusing on the quality of life of patients with MASH revealed that this particular category has an impaired quality of life due to symptoms like fatigue, generalized anxiety disorder, and bloating, all of which influenced their emotional and functional status [117]. Taking into consideration the fact that there are no approved evidence-based therapies for MASH patients, we can assume that lifestyle changes are even more challenging to implement given their disease burden; this study highlights the importance of further research to understand, characterize, and improve patients’ quality of life to decrease their disease burden.

It is generally accepted by all guidelines that dietary changes that lead to weight loss are the cornerstone primary change in MASLD patients; although the American Association for the Study of Liver Disease (AASLD) has not made specific dietary recommendations, the European Association for the Study of the Liver (EASL) recommends a diet composed of macronutrients that are in line with the Mediterranean diet (MedDiet). Based on this premise, a group of researchers conducted a multicentre, prospective randomized trial that included patients with metabolic syndrome and MASLD who followed a structured six-month lifestyle-changing treatment based on the Mediterranean diet and physical activity. The study revealed that strict adherence to the MedDiet improved intrahepatic fat content, BMI, and blood pressure, and decreased insulin resistance, underlying the importance of adherence to lifestyle changes [118]. Another similar study, this time on sarcopenia patients, revealed that adherence to MedDiet was associated with a lower risk of sarcopenia (quantified by muscle strength through handgrip dynamometry) [119]. However, studies are warranted in order to characterize the benefits of MedDiet for sarcopenia; this particular study reinforces the idea that adherence to lifestyle intervention promotes better outcomes in this category of patients. This study also pointed out that patients with low adherence to the diet were prone to an unhealthy lifestyle (being overweight or obese, smoking, consuming alcohol, and being physically inactive).

Despite the extensive research on lifestyle interventions in MASLD and sarcopenia treatment, there are numerous details to be considered when forming a treatment plan for this category of patients; guidelines right now cannot accurately recommend a specific mix of macronutrients as a diet plan, since we still do not know if a specific combination is better than another; similarly, there are currently no specific exercise regimens recommended for either MASLD or sarcopenia patients. These disparities can also contribute to a general distrust and low adherence to these general recommendations. However, taking into account all data presented, evidently, there is a crucial need for physicians to understand and incorporate the patient’s needs and expectations into their current therapy regime in order to improve their treatment response.

## 5. Conclusions

In conclusion, sarcopenia in the context of MASLD is a complex issue necessitating a multifaceted approach. Our review underscores the evolving understanding of sarcopenia’s connection with BMI and its preference for weight-adjusted indices. It explores the key mechanisms underlying the interaction between sarcopenia and MASLD, which include insulin resistance, chronic inflammation, oxidative stress, and interorgan communication facilitated by the release of cytokines (hepatokines, adipokines, and myokines).

Lifestyle interventions, particularly dietary adjustments, and tailored exercise regimens emerge as crucial strategies, although challenges persist. Bariatric surgery, alongside comprehensive lifestyle modifications, offers promise, emphasizing the need for ongoing research, patient education, and support in managing sarcopenia in MASLD patients.

## Figures and Tables

**Table 2:** Other emerging mechanisms and therapeutic potential for sarcopenic obesity.

Medication/Approach	Mechanism of Action	Potential Benefits	Limitations	Supporting Studies
**Mitochondrial Uncouplers**	Target mitochondria to enhance electron flow and ATP synthesis	Reduce adiposity while preserving lean mass; enhance energy expenditure	Efficacy in larger mammals and humans unknown; structural optimization needed	Studies on mitochondrial uncouplers such as BAM15 and SHC517 [62,63,64] suggest their potential to reverse adiposity while preserving lean mass
**S1P Receptor Agonists**	Downregulate S1P receptors and degrade sphingolipids	Increased lean mass and strength in animal models; reduced ceramide accumulation	Uncertain efficacy in aged individuals; potential inflammatory responses	Studies on Fingolimod showed increased lean mass and strength in mice with obesity [65]
**AMPK Agonists**	Stimulate AMPK, promote mitochondrial function and antioxidant capacity	Potential improvement in muscle function, reduced insulin resistance	Efficacy and side effects in humans still under research	Studies involving resveratrol activation of the AMPK pathway showed benefits in muscle function and mass in animal models [66]
**Glutathione Agonists (GlyNAC)**	Increase glutathione, an endogenous antioxidant protecting against oxidative stress	Improved muscle function and mitochondrial function in animal models and clinical trials	Long-term effects and larger population studies needed	GlyNAC supplementation demonstrated significant improvements in muscle function, gait speed, and strength in clinical trials [67]

Abbreviations: S1P: sphingosine-1-phosphate; AMPK: AMP-activated protein kinase; GlyNAC: glycine and n-Acetylcysteine.

**Table 3:** The mechanisms of muscle reconditioning facilitated by bariatric surgery.

Pathway	Outcome	Pathophysiology	Impact on Sarcopenia
Mechanical	Weight-loss	↓ mechanical stress on joints and muscles	Facilitates mobility, physical function, and rehabilitation regimens
Metabolic	Improved insulin resistance	↓ intramuscular insulin resistance ↓ myosteatosis ↑ protein synthesis	↑ muscle function and strength, irrespective of muscle mass
Inflammation	Resolution of low-grade pro-inflammatory status	↓ adipokines ↓ inflammatory markers ↓ inflammation-induced muscular catabolism ↑ protein synthesis	↑ muscle mass, contractile function

Explanations: ↓ decrease; ↑ increase.
